# The relationship between moral judgment ability, parenting style, and perfectionism in obsessive–compulsive disorder patients: A mediating analysis

**DOI:** 10.3389/fpsyg.2023.1133880

**Published:** 2023-02-27

**Authors:** Jiacheng Cui, Kongmei Zhu, Jianglin Wen, Wanjie Nie, Dong Wang

**Affiliations:** ^1^Department of Applied Psychology, Binzhou Medical University, Yantai, China; ^2^Third Hospital of Beijing Chaoyang, Beijing, China; ^3^Department of Clinical Psychology, Beijing Chaoyang Hospital, Beijing, China

**Keywords:** obsessive–compulsive disorder, parenting style, moral judgment, maladaptive perfectionism, family environment

## Abstract

**Introduction:**

Guilt is an important part of obsessive–compulsive disorder. The abnormal moral cognition of obsessive–compulsive disorder patients may be closely related to their high level of guilt. The purpose of this study was to explore the development level of moral judgment in patients with obsessive–compulsive disorder and the role of parenting style and perfectionism in moral judgment development.

**Method:**

A cross-sectional study was conducted in the clinical psychology department of a Class III hospital in Beijing. The patients with obsessive–compulsive disorder were recruited, and the healthy control subjects were recruited at the same time. Questionnaires were used to collect data, including the Yale-Brown Compulsion Scale, the Moral Judgment Test, the Parenting Style Evaluation Scale, and the Frost Multidimensional Perfectionism Scale.

**Result:**

A total of 231 patients with obsessive–compulsive disorder and 246 healthy controls were included. The results showed that, first, the obsessive–compulsive group scored significantly lower on moral judgment than the healthy control group. Second, the tendency of non-adaptive perfectionism was significantly higher in the obsessive–compulsive group than in the healthy control group. Third, parents’ excessive control, denial, punishment, and other parenting styles and non-adaptive perfectionism are higher than those of healthy people. Fourthly, the mother of obsessive–compulsive disorder patients is overly interference and protective. Rejection, denial, punishment, harshness, and father’s rejection and denial play a partial mediating role in moral judgment ability through the degree of non-adaptive perfectionism.

**Conclusion:**

The development level of moral judgment ability of patients with obsessive–compulsive disorder was significantly lower than that of the normal group, and the level of non-adaptive perfectionism was significantly higher than that of the normal group. Parents of obsessive–compulsive patients use more high-pressure control education. Parenting style partially affects the moral judgment of obsessive–compulsive patients through the degree of non-adaptive perfectionism.

## 1. Introduction

Obsessive–compulsive disorder (OCD) is a kind of psychological disease with unknown etiology, complex symptoms, poor prognosis, and serious functional impairment, which brings great mental pain to individuals and seriously affects their social life. The main performance of OCD is obsessive thinking or compulsive behavior, the main feature is the simultaneous existence of conscious self-compulsion and counter-compulsion (Veale and Roberts, 2014). OCD not only seriously destroy normal lives of patients, but the incidence of OCD also presents the tendency of increase. Many researches had suggested that OCD has become the fourth most common mental disorder after depression disorder, alcohol dependence and phobias (De Putter and Koster, 2017). In the globe, the prevalence of OCD is 2% ~ 3% (Huang et al., 2019). The further studies found most OCD patients did not seek medical or psychological treatment in the psychiatric and psychological departments of hospitals (Boger et al., 2020).

As a kind of mental disease with prolonged course, poor therapeutic effect, and poor prognosis, it is very important to deepen the understanding of its pathological mechanism for the clinical treatment of OCD. Shame and guilt are considered to be closely related to OCD (Cândea and Szentagotai-Tătar, 2018). Shame is considered a painful emotional experience associated with personal frustration or transgressive (unethical, illegal) experiences. Guilt refers to the negative emotional experience generated when an individual’s thoughts or behaviors contradict his own moral standards and values (Aardema and Wong, 2020). Previous studies have confirmed that shame and guilt are related to a variety of mental diseases such as compulsion, anxiety and depression (Olatunji et al., 2011). According to the above definition and previous studies, immorality caused by thoughts or behaviors can further trigger individual shame and guilt (Fergus et al., 2010). Therefore, moral-related research is also considered an important part of the research field of OCD. The study of Belin-Rauscent et al. showed that high moral evaluation criteria can predict the onset of OCD to a certain extent, indicating that harsh moral judgment may play a role in the onset of OCD (Belin-Rauscent et al., 2016). Jin Hong et al. found that compared with the normal group, patients with OCD generally have a strong sense of morality and a higher aversion to immoral words and deeds (Hong et al., 2016). Alonso et al. also found that the strength of moral disgust was positively correlated with obsessive–compulsive symptoms, and further speculated that “moral disgust” could be one of the important targets of clinical psychotherapy (Alonso et al., 2004).

Thus, it can be seen that the abnormal moral sense plays a crucial role in the initiation and maintenance of OCD. At present, the research in the moral field of patients with OCD mainly focuses on moral standards and moral aversion. Patients with OCD have higher moral standards and stronger sense of moral aversion. However, the level of moral judgment in patients with OCD has been poorly elucidated. Moral judgment is a comprehensive cognitive ability. German scholar Linde combined Kohlberg’s views to define moral judgment, that is, “moral judgment is the ability of individuals (based on inner moral principles) to make decisions and judge what is moral, and to act according to these judgments.” It can be seen that the ability of moral judgment reflects the level of moral cognition and moral emotion of an individual. It is the ability of an individual to independently distinguish and integrate various moral emotions and make judgment on the basis of rationality. Therefore, the low development level of moral judgment is very likely to be the cause of the abnormal moral code, moral aversion and other factors. Do people with OCD have problems with the development of this mental ability? This has never been explained.

Another important question is, if people with OCD have abnormalities in their ability to make moral judgments, what might be causing them and how? We need to look for possible answers from the research on the psychopathological mechanism of OCD. The psychopathological mechanism of OCD is very complex and the cause is not clear. At present, the research in this field mainly focuses on childhood trauma, personality characteristics and family environment (Belin-Rauscent et al., 2016; Palardy et al., 2020).

Trauma-related studies have found that childhood traumatic experiences, such as emotional abuse, emotional neglect, and physical abuse, can have long-term adverse effects on children’s neurophysiological and psychological development. Boger et al. found that the majority of OCD patients reported the above-mentioned traumatic childhood experiences, and Alonso et al. studied that OCD was related to physical and sexual abuse in childhood (Alonso et al., 2004; Boger et al., 2020).

Parents’ personality characteristics and parenting style are the decisive factors of family environment, which are the focus of researchers. Hoover’s research found that many OCD patients reported having parents with personality problems, demanding and perfectionist mothers, which led to excessive involvement and coercive parenting (Hoover and Insel, 1984).

Studies of personality traits suggest that intrusive and recurring thoughts in patients with OCD are related to the personality of the self. Doron believes that the affective structure is a risk factor for individuals to develop obsessive–compulsive disorder (Doron and Kyrios, 2005). The research of Stanley et al. shows that self-distrust and insecurity are easy to cause obsessive–compulsive symptoms, and most obsessive–compulsive symptoms are caused by self-doubt and lack of certainty cognition about things (van Leeuwen et al., 2020), and the research of Yan Jun and Cui Yuhua et al. also showed that patients with OCD have a high degree of self-disharmony and lack of flexibility and rigid personality characteristics (Yan and Cui, 2004).

Although researchers have found many meaningful results in these different aspects, most researchers believe that OCD is the result of the interaction of multiple psychosocial factors (Kong et al., 2020). Therefore, to establish a model to explain the reasons for the abnormal development of mental function in patients with OCD, we can better explain our questions. As mentioned above, this study selects moral judgment ability as the entry point, attempts to explore its role in the psychopathological mechanism of OCD, and attempts to analyze what causes the abnormal development of this function in patients. Parenting style may be an important influencing factor. A summary of previous research is not hard to find, family environment is considered to have a key impact on the formation of personality, and parenting style is the core factor of family environment (Wei et al., 2012). As the main guide in the process of individual growth, parents’ parenting style will have a great influence on their children’s psychology and behavior. Study found that the parents’ bad parenting style, such as refusal, denial, excessive interference, will lead to children’s self-denial, helplessness, inferiority, emotional depression, low sense of self-worth and other bad psychology, easy to form hypochondriasis, depression, psychopathy, mental breakdown, and other personality characteristics (Junfeng and Changguo, 2015). Therefore, the poor parenting style, through the influence of personality characteristics, and further affect individual psychological development, resulting in abnormal psychology, is very likely to be an important psychological approach to the onset of common mental diseases.

Personality traits also play a powerful role in the development of abnormal psychology. Personality traits are considered to be closely related to mental illness. A large number of studies have found that personality traits have an impact on abnormal psychology and behavior through mediating effects. For example, Ma Hongxiu et al. found that among college students, individuals with poor interpersonal relationship are prone to have suicidal ideation within 2 weeks, and the impulsive personality traits play a partial mediating role (Ma et al., 2022). In the study of Sun Ning et al., personality traits played a mediating role in the relationship between early traumatic memory and the severity of depression in the first-episode depression group (Sun et al., 2016). The relationship between personality traits and obsessive–compulsive disorder (OCD) has also attracted the attention of many researchers. OCD is considered to be one of the most closely related psychological diseases with personality traits (Zi and Zhou, 2006). Similarly, personality traits also play an important role in the onset of OCD. Xu Tingting et al. found that there was a significant positive correlation between early abuse and obsessive–compulsive symptoms, and neuroticism played a complete mediating role (Tingting et al., 2017). Among the personality traits, perfectionism is believed to be the most closely related to obsessive–compulsive disorder. Perfectionism is a personality characteristic that tends to be perfect and avoids defects and failures. Early studies on perfectionism were relatively general, and a high level of perfectionism is believed to be associated with a variety of mental diseases (Vanzhula et al., 2021). With the deepening of research, perfectionism is divided into adaptive and non-adaptive parts. In the research of abnormal psychology, non-adaptive perfectionism has received more attention. For example, studies by Huqiang Chen and others have shown that negative perfectionism plays a mediating role between attachment and obsessive–compulsive symptoms in adults (Huqiang and Lingfeng, 2017).

In conclusion, we hope to explain the psychopathological mechanism of OCD through parenting style, perfectionism tendency, and moral judgment ability. The purpose of this study is as follows: First, we want to explore whether there is any abnormality in the moral judgment ability of patients with OCD compared with the normal population. Second, we further analyzed the relationship between parenting style, perfectionism tendency and moral judgment ability in patients with OCD, and tried to explain its role in the pathogenesis of OCD. Based on the previous elaboration, we hypothesize that people with OCD develop lower levels of moral judgment than the normal population, and parenting style acts on the development of moral judgment through maladaptive perfectionism.

## 2. Materials and methods

### 2.1. Participate

From January to October 2021, a total of 149 patients with obsessive–compulsive disorder in the outpatient department of psychology of a Class three Grade A hospital in Beijing were randomly selected as the research group by convenience sampling. Each patient was recruited by two psychiatrists with more than 10 years of experience in psychiatry according to the Brief International Psychiatric Interview (Mini International Neuropsychiatric Interview; Sheehan et al., 1998). The screeners asked about depressive mood, mania/hypomania, panic attacks, agoraphobia, social anxiety, obsessive–compulsive symptoms, generalized anxiety, history of traumatic events, psychotic symptoms, anorexia, bulimia, and binge eating. Participants were also asked about their mental health, psychotherapy, and medication history. Unlike most previous studies, we included participants who did not receive psychotherapy because it was believed that receiving psychotherapy would lead to an impression of perfectionism and moral judgment. In sum, we adopt the (1) Inclusion criteria of study group: ①It meets the diagnostic criteria of DSM-5 obsessive–compulsive disorder (Kupfer, 2015), ②The age ranges from 18 to 65 years, ③The Yale-Brown Obsessive–Compulsive Scale (Y- BOCS) score ≥ 16, or the total score of Obsessive thinking or Obsessive behavior items ≥10 (Xu and Zhang, 2006), and ④Volunteer to participate in this study and sign the informed consent and (2)exclude criteria of OCD group: ① combine the other mental disorders, ②combine serious physical disease, and ③received psychological treatment or counseling before enrollment.

### 2.2. Materials

#### 2.2.1. MJT Chinese standard edition

MJT was compiled by Lind et al. In the Chinese standard edition, two moral dilemmas stories were selected: “The Foreman’s Storm” and “the Doctor’s Dilemma.” Wu Huihong et al. verified that the construct validity of the Chinese standard version of MJT was 0.835, with good reliability and theoretical validity (Yang and Wu, 2006). C score is the main indicator of moral judgment ability in MJT, with a total score of 1–100. According to Cohen’s research results, C score and moral judgment ability are divided into four grades: 1–9 is low, 10–29 is medium, 30–49 is high, and ≥ 50 is very high.

#### 2.2.2. Egma Minnen av. Bardndosnauppforstran (EMBU)

EMBU was compiled by Perris et al., Sweden, and was introduced into our country after the revision by Yue et al. (1993). The scale objectively evaluated the parenting style by asking the subjects to recall the past. The dimensions of the parenting style included six factors: emotional warmth and understanding (F1), punishment and severity (F2), excessive interference (F3), preference (F4), rejection, denial (F5), and overprotection (F6). The dimensions of maternal parenting style include five factors: emotional warmth and understanding (M1), excessive interference and protection (M2), rejection and denial (M3), punishment and severity (M4), and preference for subjects (M5). The scale consists of 66 questions, each of which is graded at 4 levels. The homoconfiguration reliability and split-half reliability of 11 EMBU factors were 0.46–0.88 and 0.50–0.91, respectively. The test–retest reliability after 3 months was 0.58–0.82.

#### 2.2.3. The Chinese version of Frost’s multidimensional perfectionism scale (FMPS-C)

FMPS-C was compiled by Frost and revised into the Chinese version by Zi and Zhou (2006). The scale included five dimensions: fear of making mistakes (CM), doubt of action (DA), organization (OR), personal standards (PS), and parental expectations (PE), with a total of 27 questions. Each question was graded by 5 levels. According to previous studies, FMPS-C can be divided into two dimensions, among which the organizational dimension belongs to the adaptive dimension and the other four dimensions belong to the maladaptive dimension. In this study, the score of maladaptive dimension was taken as the standard. The higher the score, the more severe the degree of perfectionism. The reliability of homogeneity coefficient of the total scale was 0.82, and the reliability of homogeneity coefficient of each dimension was 0.60 ~ 0.86 (Yuan et al., 2019).

### 2.3. Data analyze plan

Demographic data of the two groups were collected, including age, sex, education level, marital status, whether you are an only child, and family lived. The questionnaire was distributed by psychiatrists with years of working experience and above. They were trained before distributed the questionnaire, and the instructions and distribution methods were unified. The subjects were informed to answer the questionnaire on site and collect the questionnaire. A total of 100 questionnaires were distributed in the study group, and 84 were valid with an effective recovery rate of 84.0%. In the control group, 100 questionnaires were distributed and 87 valid questionnaires were recovered, with an effective recovery rate of 87.0%. The valid data were input into Microsoft Excel worksheet and then imported into SPSS 17.0 Chinese version for data analysis. Enumeration data were expressed as frequency and percentage (%), and comparison between groups was performed by chi-square test. After Bartlett test for homogeneity of variance and Kolmogorov–Smirnov test for normality, measurement data showed homogeneity of variance and approximately normal distribution, and were expressed as mean ± standard deviation. Independent sample t-test was used for comparison between two groups, and one-way analysis of variance was used for comparison between multiple groups. Pearson correlation was used to analyze the correlation between parental rearing style and perfectionism and moral judgment ability in patients with OCD. Multiple linear regression analysis was used to analyze the relationship among parenting style, perfectionism, and moral judgment ability of OCD patients. In a two-sided test, *p* < 0.05 indicates statistical significance.

## 3. Results

There was no significant difference in age, gender, years of education, whether the subjects were the only child, family residence, and marital status between the two groups (*p* > 0.05), as shown in Table 1.

**Table 1:** Comparison of demographic data between the two groups.

	OCD group (*n* = 231)	Control group (*n* = 246)	*χ^2^/F*	*P*
Gender [Example (%)]				
Male	106(45.9)	130(52.8)	1.673	0.196
Female	125(54.1)	116(47.2)		
Marital status [example (%)]				
married	152(65.8)	153(62.2)	0.391	0.530
unmarried	79(34.2)	93(37.8)		
Only child [example (%)]				
Yes	127(55.0)	111(45.1)	2.120	0.146
No	104(45.0)	135(54.9)		
Place of Residence of family [example (%)]				
urban	173(74.9)	177(72.0)	0.152	0.701
countryside	58(25.1)	69(28.0)		
age (years, $\bar{x}\pm s$ )	30.0 ± 9.0	30.6 ± 9.2	0.192	0.988
Years of education (years, $\bar{x}\pm s$ )	13.4 ± 2.1	13.5 ± 1.8	0.014	0.839

Comparison of the scores of EMBU, FMPS-C, and MJT Chinese standard edition between the two groups: the scores of F2, F3, F5, F6, M2, M3, and M4 in EMBU of the study group were higher than those of the control group, and the differences were statistically significant (*p* < 0.05). The scores of FMPS-C maladaptive dimension in the study group were higher than those in the control group, and the scores of FMPS-C adaptive dimension and MJT Chinese standard version were lower than those in the control group, and the differences were statistically significant (*p* < 0.01). These results are shown in Table 2.

**Table 2:** Comparison of EMBU, FMPS-C, and MJT Chinese standard edition scores between the two groups (scores).

	OCD group (*n* = 231)	Control group (*n* = 246)	*F*	*P*
EMBU				
Paternal parenting style				
F1’ scores	48.8 ± 9.1	49.7 ± 12.3	0.251	0.616
F2’ scores	29.1 ± 7.6	16.3 ± 6.1	137.295	<0.001
F3’ scores	25.3 ± 4.7	17.1 ± 5.0	23.762	<0.001
F4’ scores	6.5 ± 5.2	6.3 ± 5.8	0.026	0.872
F5’ scores	15.4 ± 4.2	8.7 ± 3.4	93.649	<0.001
F6’ scores	15.4 ± 2.8	9.3 ± 3.1	33.363	<0.001
Maternal parenting style				
M1’ scores	49.0 ± 8.3	50.2 ± 8.0	0.741	0.322
M2’ scores	43.2 ± 4.7	34.5 ± 5.0	79.921	<0.001
M3’ scores	16.2 ± 4.9	11.9 ± 4.1	26.273	<0.001
M4’ scores	14.9 ± 5.8	11.7 ± 5.2	5.993	0.021
M5’ scores	5.3 ± 4.8	6.1 ± 5.3	1.536	0.294
FMPS-C				
Adaptive dimension scores	17.3 ± 5.1	24.1 ± 6.0	78.571	<0.001
Maladaptive dimension scores	75.12 ± 10.4	57.59 ± 9.7	115.395	<0.001
Score of MJT Chinese standard edition	8.8 ± 2.0	15.2 ± 5.9	66.527	<0.001

EMBU Parenting Style Evaluation Scale; M1 emotional warmth and understanding; M2 excessive interference and protection; M3 Reject, deny; M4 punishment, severe; M5 prefers subjects; F1 emotional warmth and understanding; F2 punishment, severity; F3 excessive interference; F4 prefers subjects; F5 Reject, deny; F6 overprotection; FMPS-C Frost Multidimensional Perfectionism Scale Chinese version; MJT Moral Judgment Test.

M2, M3, and M4 in parenting style were positively correlated with perfectionism (*p* < 0.01), and negatively correlated with moral judgment (*p* < 0.05). F2, F5, F3, and F6 were positively correlated with perfectionism (*p* < 0.05), and F5 was negatively correlated with moral judgment (*p* < 0.01). See Tables 3, 4.

**Table 3:** Correlation between maternal parenting style and perfectionism and moral judgment ability of OCD patients (r value).

	**Maternal parenting style**				
	M1	M2	M3	M4	M5
Level of maladaptive perfectionism	−0.051	0.899^a^	0.551^a^	0.419^a^	0.189
Moral judgment ability	0.063	−0.838^a^	−0.291^b^	−0.375^a^	−0.179

M1 emotional warmth and understanding; M2 excessive interference and protection; M3 Reject, deny; M4 punishment, severe; M5 prefers subjects; F1 emotional warmth and understanding.

**Table 4:** Correlation between parental rearing style and perfectionism and moral judgment ability of OCD patients (r value).

	Paternal parenting style					
	F1	F2	F3	F4	F5	F6
Level of maladaptive perfectionism	−0.049	0.304^a^	0.233^b^	0.118	0.690 ^a^	0.282^b^
Moral judgment ability	0.084	−0.131	−0.185	−0.092	0.690 ^a^	−0.092

F2 punishment, severity; F3 excessive interference; F4 prefers subjects; F5 Reject, deny; F6 overprotection; a: *P* < 0.01; b: *P* < 0.05.

According to the mediating principle, M2, M3, M4, and F5 were included in the mediating effect analysis because they were related to both the degree of maladaptive perfectionism and the ability of moral judgment, respectively. Three regression equations were established: (1) The unitary regression equation of the influence of parenting style (X) on moral judgment ability (Y); (2) Unitary regression equation of the influence of parenting style (X) on the degree of maladaptive perfectionism (M); (3) The binary regression equation of the influence of parenting style (X) and degree of maladaptive perfectionism (M) on moral judgment ability (Y). M2, M3, M4, and F5 in EMBU were included in the three equations for analysis, and the four factors were all *P* value in the unitary regression analysis. 0.05, as shown in Table 5. When the degree of maladaptive perfectionism entered the equation and became a binary regression equation, the four factors were still *P* value 0.05, but β values decreased, as shown in Table 6. These results indicate that the four factors in EMBU have a partial mediating effect on moral judgment ability through the degree of maladaptive perfectionism.

**Table 5:** Multiple linear regression analysis of the influence of parenting style on moral judgment ability and degree of maladaptive perfectionism in patients with OCD.

Dependent variable	Independent variable	*β*	*S.E.*	*B*	*t*	*P*
Moral judgment ability	Parenting style					
	M2	−0.717	0.045	−0.374	−9.816	<0.001
	M3	0.307	0.025	0.101	5.013	<0.001
	M4	−0.284	0.021	−0.149	−4.196	<0.001
	F5	−0.251	0.038	−0.103	−3.793	<0.001
Degree of maladaptive perfectionism	Parenting style					
	M2	0.798	0.085	1.837	17.392	<0.001
	M3	−0.089	0.062	−0.131	−2.039	0.048
	M4	0.185	0.051	0.175	2.998	0.006
	F5	0.129	0.112	0.249	2.441	0.017

Excessive interference and protection of M2; M3 Reject, deny; M4 punishment, severe; F5 Reject or deny.

**Table 6:** Multiple linear regression analysis of the effects of maladaptive perfectionism and parenting style on moral judgment ability in patients with OCD.

Variable	*β*	*S.E.*	*B*	*t*	*P*
Parenting style(X)					
M2	−0.504	0.041	−0.243	−6.492	<0.001
M3	0.207	0.015	0.063	5.414	<0.001
M4	−0.075	0.010	−0.021	−1.992	0.024
F5	−0.113	0.018	−0.058	−3.341	0.004
Degree of maladaptive perfectionism(M)	−1.294	0.028	−0.029	−15.391	<0.001

Excessive interference and protection of M2; M3 Reject, deny; M4 punishment, severe; F5 Reject or deny.

## 4. Discussion

The purpose of this paper is to discuss the possible role of parenting style, perfectionism tendency and moral judgment ability in the psychological pathogenesis of OCD through questionnaire survey and statistical analysis. First of all, we want to explore whether there is any abnormality in the moral judgment ability of patients with OCD compared with the normal population. We hypothesized that the level of moral judgment in patients with OCD was significantly lower than that in the normal population. In the MJT test, two “moral dilemma stories” were used to test the development level of moral judgment in patients. In 1958, Kohlberg initially put forward the theory of the development of individual moral judgment. Kohlberg’s moral theory is divided into three levels. At the pre-custom level, individuals judge whether the event is moral or not based on whether the outcome of the event is in line with personal interests, the criterion of individual judgment in this stage is immoral. At the custom level, the individual’s judgment is still based on the outcome of the event, but the authority and social norms can be used as a measuring scale to judge whether the event is moral or not. At the post custom level, individuals not only pay attention to the outcome of events, but also attach importance to the analysis of event motives, they are able to realize that even social norms are not immutable (Yu and Liu, 2011). Through further analysis of OCD patients’ answers to questions, it is found that patients may make moral judgments mainly based on whether the event results conform to social norms and laws. First, in both of the dilemma stories, patients had lower approval ratings for the protagonist’s behavior. Then, in the disapproving concept part, patients had a high degree of agreement with items such as “breaking the law,” “violating social order,” and “violating professional norms.” Therefore, it can be inferred that compared with the normal group, the moral judgment ability of OCD patients may be at the level of custom stage, and it is difficult to combine motivation to think about individual behavior. As mentioned above, previous studies have mainly found that OCD patients have higher moral judgment labeling and stronger moral aversion, This result may complement previous research in the field of morality in OCD patients. OCD patients may have a lower level of moral cognition and a more absolute and rigid judgment standard than normal people, so they have a more rigorous moral judgment on events and are prone to have a stronger sense of moral aversion. There are few previous studies on the moral judgment ability of patients with obsessive–compulsive disorder. To some extent, this study provides a new theoretical support for the research on the moral field of OCD.

In defining moral judgment ability, Professor Linde adopted the definition used by Kohlberg, that is, “Moral judgment is the ability of an individual (based on inner moral principles) to make decisions and judgments about what is moral, and the ability to act on those judgments.” The ability of moral judgment includes three aspects: moral cognition, moral behavior and moral emotion. At the same time, Professor Linde proposed that “moral cognition is the ability of moral judgment, the ability of individuals to distinguish and integrate various moral emotion independently and make judgments on the basis of rationality,” while “moral emotion are individuals’ attitudes toward moral concepts, values and morals” (Lind, 1984). It was also suggested that moral judgment is a comprehensive ability based on the moral cognition and further guide individual moral behavior (Yang and Wu, 2006). Education influences the formation and development of moral cognition and moral emotion, thus affecting moral judgment ability. Under the influence of a highly controlled family environment, OCD patients are constantly strict with themselves, hoping to meet their parents’ expectations of them, thus showing excessively high moral standards, and giving strong moral color to “immoral events” considered by some normal groups (such as the random thoughts of attacking others when angry). It can be seen that OCD patients have abnormal cognition of moral standards. In combination with previous research, people with OCD tend to hold themselves and others to an unreasonably high moral standard to judge their thoughts and actions. This standard is often maladaptive, meaningless, and makes patients feel extremely painful, while the patients are difficult to adjust by themself, and still use similar cognition to make moral judgment. Therefore, compared with healthy people, OCD patients show a low level of moral judgment ability.

Secondly, research on personality traits has found that people with OCD are more perfectionist than healthy people. Further analysis found that the score of the maladaptive dimension of OCD patients was significantly higher than that of the control group, while the score of the adaptive dimension was significantly lower than that of the control group. This suggests that high levels of maladaptive perfectionism might be an important feature that distinguishes OCD patients from the normal group.

Perfectionism is a kind of personality trait, and it is the psychological tendency to pursue the perfection of anything possessed by personality. The core characteristics of perfectionism are setting a unique high standard, constant self-criticism and self-reflection, fear of failure, attention to appearance, and pursuit of order and neatness. Studies have shown that perfectionism is a high risk factor for many physical and mental diseases, including OCD (Wang and Lang, 2005). Since the 1980s, with the deepening of research, the multi-aspects of perfectionism have been gradually explained. Perfectionism has both adaptive and maladaptive dimensions. Among them, the research found that the high standards of adaptive perfectionism are relatively reasonable and come from the self, and the pursuit of high standards is accompanied by the improvement of self-worth, sense of meaning, sense of accomplishment, self-satisfaction, and self-esteem. However, maladaptive perfectionism leads individual to make excessive, rigid standards, while behind show the fear of making mistakes as well as to their doubt of ability, lead to patients addicted to high standards, constantly cannot follow the changes in the environment and adjust, and difficult to gain pleasure from record of (Williams and Levinson, 2021). The cognitive schema of maladaptive perfectionists can cause, promote, and maintain persistent tension and stress, thus leading to psychological disorders, and is also believed to be closely related to many negative psychological experiences (such as tension, worry, embarrassment, etc.) and psychological problems (Sametoğlu et al., 2022). The result of the level of perfectionism in this study is consistent with previous research, for example, Taylor et al. found that the level of maladaptive perfectionism in obsessive–compulsive patients was significantly higher than that in depressed, anxious, and normal groups, and was closely related to insecure attachment (Taylor et al., 2017). Zakiei et al. found that the level of maladaptive perfectionism in patients with OCD was correlated with the severity of obsessive–compulsive disorder (Zakiei et al., 2017).

Thirdly, we found that parents of people with OCD tend to use methods like excessive interference and protection, refusal and denial, criticism, punishment, and so on. Factors M2, M3, and M4 of maternal parenting style and F2, F3, F5, F6 of paternal parenting style were positively correlated with maladaptive perfectionism. This is also consistent with previous research. For example, Zhao et al. found that the intimacy and understanding degree of family parents in the OCD group was significantly lower than that in the control group, while the degree of parental punishment, interference and control, as well as family contradiction was significantly higher than that in the control group. It is not difficult to see that maladaptive perfectionism has close relationship with this rigorous and high-pressure parenting style. The parents of OCD patients always try to constant intervention in the hope of gaining more control over their children’s lives. At the same time, parents tend to set complicated life norms and strict achievement goals for their children. When patients does not meet requirements, their parents will give severe criticism and punishment, but when the patient achieves the goal, the patient’s parents deny the achievement, think the patient should do better, and set further goals. This leads to the patient being cautious, easy to blame, excessive pursuit of “perfection,” resulting in tension and fear, and further lead to their inferiority and depression (Shuanghu and Kai, 2010). Previous studies have found that such parenting style of parents of OCD patients cultivates high levels of stigma, guilt and self-condemnation in their children, which provides sufficient conditions for the onset of neurosis (Wang, 2016).

Finally, in the mediation model analysis, according to the principle, we included four parenting factors M2, M3, M4, and F5 which are related to both maladaptive perfectionism and moral judgment ability. In the univariate regression analysis, parenting style had a significant effect on maladaptive perfectionism and moral judgment. Through further binary regression analysis, it can be found that maladaptive perfectionism plays a partial mediating role between the four included factors and moral judgment ability. Previous studies have shown that adverse personality traits may mediate the relationship between home environment factors and abnormal mental functioning in patients with mental illness (Raines et al., 2019). This indicates that in the OCD group, the characteristic parenting style is an important path through shaping the personality characteristics and further affecting the abnormal development of patients’ psychological function. At the same time, it can be seen that three factors of maternal upbringing were included in the regression analysis, while only one factor of paternal upbringing was included, which may indicate that mothers play a greater role in the formation of patients’ abnormal psychology. As the main caregiver of an individual, the poor mental health of the mother and the adoption of low warmth, denial, and strict education methods will lead to poor mother–child interaction and the whole family dynamics, which increases the possibility of the individual suffering from schizophrenia, depression, neurosis, and other mental diseases (Abbaspour et al., 2021).

As mentioned above, people with OCD have high levels of shame and guilt, and in order to avoid these painful experiences, they strive for a “flawless” state. Studies have shown that the areas that people with OCD are overly concerned about are also the areas that their parents are most concerned about, such as “moral character,” “academic achievement,” and “cleanliness and hygiene” (Tang et al., 2010). The relatively low level of moral cognition and moral judgment of patients with OCD may be to achieve moral “perfection” and avoid shame and guilt, but the result is not satisfactory. When the judgment standard is improved, more and more thoughts or behaviors are included in the category of “immoral,” which aggravates the shame and guilt of patients. Meanwhile, patients’ awareness of “immoral” content is also continuously improved, leading to serious anxiety. Behind these symptoms, there is no doubt the fear of “bad” and “imperfect,” which runs through the growth of people with OCD. At an early age, people with OCD have realized that if they are not virtuous, they will prove themselves to be “selfish” and “hated” and should be punished. Even as adults, they cannot accept their “immorality.”

To sum up, the excessive interference and protection, refusal to deny and punishment, and strict parenting of parents of patients with OCD are not conducive to the formation of patients’ moral judgment ability, leading to the reduction of their moral cognition and moral emotion, so when encountering stressful events, their moral behavior is also abnormal. The negative parenting style makes the patient seldom get the affirmation and support from the parents, thus forming the character of maladaptive perfectionism, and the maladaptive perfectionism also affects the moral judgment ability of the patient. The results of this paper also bring us enlightenment in clinical and family education. First of all, in the clinical aspect, the current first-line treatment of obsessive–compulsive disorder is high-dose SSRI combined with cognitive behavioral therapy(CBT), which is based on expose-response prevention technology(ERP). It is not difficult to see that the current treatment of obsessive–compulsive disorder is mainly symptom-oriented. However, this study leads us to find that moral cognition may be a starting point for the psychotherapy of obsessive–compulsive disorder. At the same time, understanding the family factors behind the symptoms is also crucial. Therefore, it is necessary to conduct in-depth individual psychotherapy and family therapy for patients with OCD, especially adolescent patients. And in terms of education, we also need to think how to adjust the parenting style, so that patients have a relaxed growth environment, so that their moral judgment ability has a good development, which is also crucial to the development of their personality.

## 5. Conclusion

We found that people with OCD had lower levels of moral judgment development and lower levels of adaptive perfectionism than healthy people, but higher levels of maladaptive perfectionism. And obsessive–compulsive parents more use excessive interference control, criticism, and denial of education. Through further analysis, it can be seen that parenting style partially mediates the moral judgment ability of OCD patients through the degree of maladaptive perfectionism.

## Data availability statement

The original contributions presented in the study are included in the article/Supplementary material, further inquiries can be directed to the corresponding author.

## Ethics statement

The studies involving human participants were reviewed and approved by Ethics Committee of Beijing Chaoyang Hospital. The patients/participants provided their written informed consent to participate in this study.

## Author contributions

JC was responsible for the study design. JC and KZ was responsible for the data collection, analysis, and article writing. JW and WN were responsible for collecting the data and writing the article. DW was responsible for the review and suggestion of the research design and article writing, and provided resource support. All authors contributed to the article and approved the submitted version.

## Conflict of interest

The authors declare that the research was conducted in the absence of any commercial or financial relationships that could be construed as a potential conflict of interest.

## Publisher’s note

All claims expressed in this article are solely those of the authors and do not necessarily represent those of their affiliated organizations, or those of the publisher, the editors and the reviewers. Any product that may be evaluated in this article, or claim that may be made by its manufacturer, is not guaranteed or endorsed by the publisher.
